# Analysis of the summer thermal comfort indices in İstanbul

**DOI:** 10.1007/s00484-024-02669-7

**Published:** 2024-04-24

**Authors:** Merve Yılmaz, Yiğitalp Kara, Hüseyin Toros, Selahattin İncecik

**Affiliations:** 1 Faculty of Aeronautics and Astronautics, Department of Meteorological Engineering, İstanbul Technical University, Maslak, İstanbul 34469 Türkiye; 2 Faculty of Aeronautics and Astronautics, Department of Meteorological Engineering, Samsun University, Ondokuzmayıs, Samsun 55420 Türkiye; 3 International Union of Air Pollution Prevention Associations-IUAPPA, 124, Sajik-ro, Jongno-gu, Seoul, Korea

**Keywords:** Regional biometeorology, Outdoor thermal comfort, Thermal indices, Summer heat stress, Mediterranean region, Urban microclimate

## Abstract

Thermal indices and thermal comfort maps have great importance in developing health-minded climate action strategies and livable urban layouts. Especially in cities where vulnerability to heatwaves is high, it is necessary to detect the most appropriate indicators for the regional characteristics and action planning with respect to thermal comfort. The aim of the study is to examine thermal indices as indicators of regional climate characteristics by relating to meteorological parameters and spatial features. Atmospheric variables including air temperature, wind speed, cloud cover, and relative humidity data were obtained from 30 meteorological stations located in districts having different climatic features. Heat stress levels for apparent temperature (AT), heat index (HI), wet bulb globe temperature (WBGT), physiological equivalent temperature (PET), universal thermal climate index (UTCI), and perceived temperature (PT) indices were calculated and associated with meteorological parameters. Thermal comfort maps have been created with the daily mean and maximum values of all indices. As a result, the meteorological parameters with the strongest correlation with all thermal indices are air temperature (T_a_) with *r* = 0.89 ± 0.01 and mean radiant temperature (T_mrt_) with *r* = 0.75 ± 0.16. The differences in thermal stress levels over the city have been distinctively observed in the AT_max_, PET_max_, and PT_max_ maps, which are generated by the daily maximum values of the indices. Çatalca, where forests cover large areas compared to highly urbanized districts, has the lowest heat stress defined by all indices.

**Supplementary Information:**

The online version contains supplementary material available at 10.1007/s00484-024-02669-7.

## Introduction

In the Sixth Assessment Report (AR6) of IPCC (Intergovernmental Panel on Climate Change), it was reported that vulnerability and risks driven by climate change and heatwave hazards have mostly increased in cities and settlements (IPCC 2022). As in many cities of the world, rapid population growth in İstanbul causes an imbalance between demand and supply, a change in land use that affects meteorological features, and inescapably causes a change in the external climate (Falasca et al. 2019). Urbanization and an increasing population have influenced the temperature records of cities and caused a strengthening of the urban heat island intensity (Tayanç and Toros 1997; Ünal et al. 2020) and increased negative effects of climate change on human health and thermal comfort (Rajan and Amirtham 2021).

Previous studies in the field of heat-health relationships have indicated that heatwaves and rising air temperatures cause increases in mortality and morbidity and pose risks to human welfare, life satisfaction, and mental health (IPCC et al. 2022; Weilnhammer et al. 2021); mostly, mortality and hospital admission rates have been directly associated with air temperatures (Basu et al. 2012; Gasparrini et al. 2015). Nevertheless, the way humans perceive air temperatures recorded by a thermometer varies due to factors such as humidity, wind, regional climate attributes, and personal circumstances, including physiological traits, working conditions, and clothing thickness. To effectively pinpoint heatwaves and their health repercussions, as well as devise strategies to alleviate these impacts, it is imperative to consider not just meteorological factors but also bioclimatic elements such as thermal stress and thermal comfort (Dimitriadou et al. 2021; Heo and Bell 2019; Jendritzky and Tinz 2009; Vaneckova et al. 2011; Yılmaz et al. 2023; Urban et al. 2019).

Meteorological factors such as temperature, humidity, wind, and solar radiation generate a thermal stress on people due to the continuous heat exchange between the human body and the atmosphere (Gosling et al. 2014; Potchter et al. 2022). The thermal environment is the space in which the human body and the atmosphere interact and the satisfaction of people with the thermal environment is called “thermal comfort”. (ANSI/ASHRAE 2010; Jendritzky and Tinz 2009). Numerous thermal indices have been created by various institutions to assess thermal stress and delineate conditions of thermal comfort or discomfort (Shooshtarian et al. 2020). These thermal indices can be broadly categorized into two groups: straightforward or direct indices, which rely solely on meteorological factors for calculation, and indices derived from the energy balance between individuals and their thermal environment (Blazejczyk et al. 2012).

Thermal indices can be a valuable tool in assisting with the achievement of the Sustainable Development Goals (SDGs) by providing useful information for monitoring, predicting, and mitigating the impacts of extreme heat on human health and well-being (Dimitriadou et al. 2021; He et al. 2019; Heo and Bell 2019; Urban et al. 2019), defining heatwaves (Yılmaz et al. 2023), developing heat warning systems (Folkerts et al. 2021), environmental evaluation of city parks or natural areas and the reconstruction of the urban landscape (Cohen et al. 2014), and supporting the transition to a more sustainable future (Nevat et al. 2021). Urban climate maps integrated with environmental parameters and thermal stress are regarded as essential for urban planning that takes care of human thermal comfort (Cetin et al. 2018; Chen et al. 2017; Moisa et al. 2022). Urban thermal comfort is affected by the interaction of meteorological conditions and underlying surface characteristics in the sub-districts (Kim et al. 2022; Roshan et al. 2020; Wang et al. 2004).

Several studies about urban thermal comfort mapping were conducted in a number of cities in Turkey with the aim of improving city plans that are sensitive to the thermal environment and determining suitable regions for tourism and recreation (Altunkasa and Uslu 2020; Cetin 2015; Cetin et al. 2019; Gungor et al. 2021; Topay 2013; Toros et al. 2005). However, thermal comfort maps should be individually created for each city, because the cities have typical land use and climate characteristics. Although, Matzarakis and Karagülle (2007) examined the thermal comfort conditions over İstanbul in terms of PET means, extremes, and frequencies; a regional analysis and mapping study of thermal comfort has not yet been carried out for the city, which has Mediterranean climate characteristics and interacts with the surrounding other climate regions (Deniz et al. 2011; Incecik 1996).

Rapid population growth, unplanned urbanization, and deforestation have led to an increase in urban heat island density in İstanbul. Temperature increases were more pronounced in the southern parts of İstanbul, where population density and urbanization are higher, compared to the northern districts (Karaca et al. 1995). İstanbul has unique climatic characteristics because of the Bosphorus Strait, which connects the Black Sea in the north and the Marmara Sea in the south (Incecik and Im 2012). Since the intensity of residential areas, access to green spaces, and proximity to the coasts vary across the districts in İstanbul, the climatic characteristics of the districts are different from each other (Ünal et al. 2020). Sub-climatic characteristics of İstanbul districts could be classified as urban or forest, coastal or inland, north or south. The detailed climatic feature categories of districts are given in the Supplementary Material, Table S1

In order to examine the effects of heatwaves on human health due to climate change and develop action policies against those effects, biometeorological parameters should be considered as well as typical meteorological variables such as temperature, humidity, air pressure, wind, precipitation, and the solar radiation. Moreover, mapping and regional analysis of climate characteristics are important issues to determine risks and take action locally. In this study, we aim to explain the relationship between thermal stress levels and meteorological parameters in consideration of micro-climate within districts and surface characteristics. To achieve this aim, we calculated the correlation coefficients between hourly measured meteorological variables and hourly calculated thermal indices in all districts of İstanbul by asking the questions of whether or to what extent an inference can be drawn about the thermal comfort of the districts by looking at the measured relative humidity and wind speed values. We also wanted to determine which indices would be appropriate for a regional thermal comfort analysis in İstanbul during the course of the summer and compared to other Mediterranean cities in terms of thermal comfort. We created thermal comfort maps with the summer means of daily average and daily maximum AT, HI, WBGT, WBGT, PET, UTCI, and PT levels observed in all districts and graded them according to the scale of the index assessments; thus, we identified the indices maps where the most significant variability between districts can be observed.

## Methodology

### Study area

İstanbul ranks as the 23rd largest metropolis globally, boasting a population of 16 million residents and covering a total area of 5,460 square kilometres. Geographically, the city straddles two continents, with the Bosphorus serving as a natural divider between Europe and Asia. To its north lies the Black Sea, while the southern border is formed by the Marmara Sea. İstanbul differs from other megacities in terms of climatic characteristics due to its unique geography (Deniz et al. 2011; Incecik and Im 2012). The relief of this territory is mostly lowland-hilly, with relatively low altitude and low values in the vertical segmentation index, therefore it does not have serious effects on its climatic features in meso-climatic terms. The main factor of the regional climate here is the proximity of the territory to large bodies of water that surround it from the north (the Black Sea), from the south (the Sea of Marmara), and through the Bosphorus Strait. This has a moderating effect on the local climate, which takes on maritime features similar to the Mediterranean climate. Although the climatic structure varies regionally due to the influence of the Marmara Sea and the Bosphorus, the city has a Mediterranean climate. Winters are typically cold and wet, while summers are hot and humid; the average temperature ranges between approximately 24 °C in summer and 5–10 °C in winter. The total annual long-term precipitation is 677 mm, observed to be intense between October and March. The predominant wind direction throughout İstanbul is NE (northeasterly) which is called Poyraz. The second most effective wind direction is N (north) direction -Yıldız- and the third strongest direction is SW (southwest) named as Lodos (Toros et al. 2017). As per İstanbul’s climatic profile, the months of June, July, and August (JJA) stand out as the hottest period of the year.

**Fig. 1:**
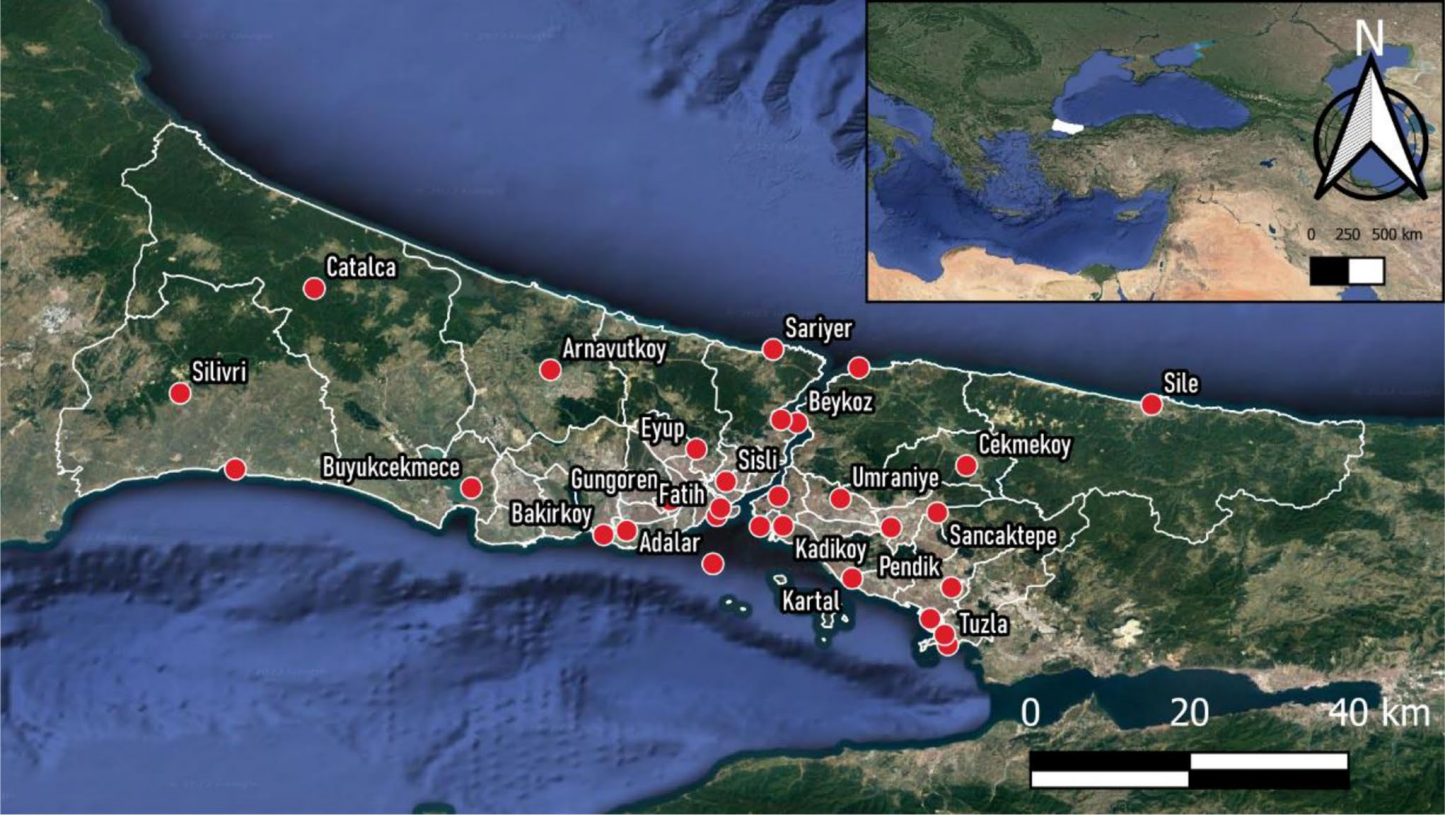
Physical-geographical map of İstanbul and locations of meteorological monitoring stations

Most of the urbanized zones hug the southern coastline and line the picturesque Bosphorus, whereas the northern regions consist of watersheds and forests. This sprawling mega city is subdivided into 39 districts, each of which is overseen by local municipalities, with 25 on the European side and 14 on the Asian side. The physical-geographical map of İstanbul with district borders is given in Fig. 1 and the CORINE land use map in Figure S1. Since the population and urbanization are denser in the south of İstanbul, temperature increases due to climate change and the urban heat island effect are higher in these regions compared to the northern regions (Ezber et al. 2007; Karaca et al. 1995; Tayanç and Toros 1997; Ünal et al. 2020). In addition, the impact of urbanization on climate is more noticeable in summer months (Ezber et al. 2007).

### Meteorological data

We collected hourly data on air temperature, relative humidity, wind speed, and cloud cover for the summer months (June, July, and August) spanning 2013 to 2017 from the Turkish State Meteorological Service (TSMS). The choice of a 5-year period, commencing in 2013, for our study was driven by the fact that prior to this timeframe, there were insufficient meteorological observation stations in İstanbul to support regional analyses. Our dataset was sourced from a network of 30 monitoring stations managed by TSMS, strategically positioned across 21 districts in İstanbul. On the European side, these districts include Adalar, Bakırköy, Beykoz, Büyükçekmece, Çatalca, Eyüp, Fatih, Güngören, Sarıyer, Silivri, Şile, Şişli, and Tuzla. On the Asian side, the districts encompass Arnavutköy, Çekmeköy, Kadıköy, Kartal, Pendik, Sancaktepe, Ümraniye, and Üsküdar. The exact locations of these meteorological stations can be observed in Fig. 1; detailed topographical and climatic features of these locations is given in Supplementary Material, Table S1

The preprocessing of the raw dataset was conducted to identify outliers, bad and missing values and to check the homogeneity of the variables. Outliers and bad data were removed and handled as missing data, and the ratio of missing data in the time series of air temperature, relative humidity, wind speed, and cloud cover recorded at each station is given in Supplementary Material, Table S1 According to the results of Levene’s Test and Bartlett’s Test, it is found that the meteorological data are not homogeneously distributed (see Supplementary Material for test results); therefore, k-nearest neighbor (kNN) algorithm, which can be used with high performance in heterogeneous datasets and allows a flexible and easy implementation with a non-parametric approach (Santos et al., 2022), was preferred to impute the missing data (Yılmaz et al. 2023). This machine learning method identifies the closest neighboring variables by measuring the variable distances, calculates a weighted average based on the nearest k neighbors, and then replaces the missing value with this computed average (Badhiye et al. 2013). We employed the Euclidean distance function with a selected k value of 10. All data analysis and the imputation of missing values were carried out using RStudio Software (v4.2.1; R Core Team 2022), with the assistance of the DMwR2 package (v0.0.2; Torgo 2016).

It is worth noting that there were no meteorological observation stations in the following 18 districts: Maltepe, Beyoğlu, Esenler, Beylikdüzü, Sultanbeyli, Başakşehir, Zeytinburnu, Avcılar, Kağıthane, Gaziosmanpaşa, Küçükçekmece, Sultangazi, Bahçelievler, Beşiktaş, Esenyurt, Bayrampaşa, Bağcılar, Ataşehir, and Sultanbeyli. To address the absence of data in these districts, we utilized hourly meteorological data from the nearest observation station, taking into account both proximity, topographic similarity and population density.

### Calculation of thermal indices

The thermal comfort levels in İstanbul were calculated hourly with six different thermal indicators from both the simple indices group and the energy balance-based indices group. Apparent temperature (AT), heat index (HI), and wet bulb globe temperature (WBGT) levels, which are simple indices, were determined by special algorithms that only used meteorological parameters. On the other hand, energy balance-based indices take into account all of the heat exchange mechanisms between the human and the thermal environment and it is required to consider physiological standards and the individual heat budget models for their calculation. The physiological equivalent temperature (PET), universal thermal climate index (UTCI), and perceived temperature (PT) levels, involved in the energy balance-based indices group, were calculated by RayMan Pro (v3.1 Beta) software. The hourly air temperature, relative humidity, wind speed, and cloud cover data were used as inputs.

RayMan has been developed to compute the mean radiant temperature (T_mrt_) and thermal indices by determining radiation fluxes in both straightforward and complex scenarios (Matzarakis et al. 2007, 2010). RayMan employs a time-independent technique for calculating these values for specific locations, enabling the processing of substantial meteorological observation data spanning multiple years. The estimation of global radiation or shading at specified coordinates relies on user-provided inputs (Matzarakis and Fröhlich 2018). It’s worth noting that T_mrt_ and thermal indices exhibit a high sensitivity to meteorological variables (Fröhlich et al. 2019).

T_mrt_ serves as an indicative surface temperature that encapsulates the combined impact of both shortwave and longwave radiation experienced by humans (Kántor and Unger 2011). While T_mrt_ was not measured directly, this study assessed it as a meteorological parameter due to its crucial role as an input in the computation of thermal indices. Hourly T_mrt_ values were further derived using RayMan Pro software.

It is an important advantage that simple indices can be easily calculated using directly measured meteorological variables without need for the T_mrt_ indicator. On the other hand, energy balance-based indices can be more explanatory, especially in studies concerning the temperature-health relationship, since they also include specific variables relevant to the human body (Yılmaz et al. 2023).

#### AT - apparent temperature

The most frequently used metric in thermal comfort and environmental health studies is the apparent temperature parameter. In order to calculate the apparent temperature, Steadman developed a set of equations that took into account a number of variables, including air temperature, radiation, wind speed, vapor pressure, and the heat resistance of clothing and skin; then standardized these equations by making specific assumptions about personal circumstances in order to calculate them using only meteorological variables (Steadman 1994). Since the radiation was not measured within the study area, the approach using temperature, vapor pressure and wind speed was adopted, other AT formulas from the same study can be found in the Supplementary Material file. The AT model takes into account both the sultriness effect of humidity at higher temperatures and the chilling effect of the wind at lower temperatures. The following equation, created by the Australian Bureu of Meteorology (ABM), was used to calculate the hourly vapor pressure values (ABM 2021):

AT = T_a_ + 0.33 P_vap_ – 0.7 WS – 4.00

$${{\rm{P}}_{{\rm{vap}}}}{\rm{ =\,\,  }}{{{\rm{RH}}} \over {{\rm{100}}}}{\rm{ }} \times {\rm{ 6}}{\rm{.105 }} \times {\rm{ exp\, }}{{{\rm{17}}{\rm{.27 }}{{\rm{T}}_{\rm{a}}}} \over {{\rm{237}}{\rm{.7  +  }}{{\rm{T}}_{\rm{a}}}}}$$

AT: Apparent temperature (°C), T_a_: Temperature (°C), P_vap_: Vapor pressure (hPa),

WS: Wind speed (m/s), RH: Relative humidity (%).

#### HI - heat index

The heat index, also known as the felt temperature, quantifies the impact of humidity on how the weather is perceived in hot conditions. It’s evident that as relative humidity levels rise, the perceived temperature also increases. The Heat Index (HI) is determined through an algorithm adapted from the original Steadman tables, as employed by the National Weather Service (NWS) of the United States’ National Oceanic and Atmospheric Administration (NOAA) (NWS 2021). These calculated values are then grouped into categories based on the potential health implications they carry (see Supplemantary Material Table S.2).

HI = c_1_ + c_2_T + c_3_R + c_4_TR + c_5_T^2^ + c_6_R^2^ + c_7_T^2^R + c_8_TR^2^ + c_9_T^2^R^2^.

HI = Heat index (°C), T = Air temperature (°C), R = Relative humidity (%).

**Table:** 

c_1_ = -8.78469475556	c_4_ = -0.14611605	c_7_ = 0.002211732
c_2_ = 1.61139411	c_5_ = -0.012308094	c_8_ = 0.00072546
c_3_ = 2.33854883889	c_6_ = -0.0164248277778	c_9_ = -0.000003582

#### WBGT - wet bulb globe temperature

WBGT was developed by the US Navy in the 1950s as part of the examination of temperature-related disturbances in military training. It has been calculated using the natural wet bulb temperature (t_nw_), globe temperature (t_g_), and air temperature (t_a_) parameters measured with wet bulb thermometers, globe thermometers, and dry bulb thermometers, respectively. (Blazejczyk et al. 2012; Bs En Iso 7243, 2017)

WBGT = 0.7 t_nw_ + 0.2 t_g_ + 0.1 t_a_.

Because of the difficulties in the measurements of globe thermometer and wet bulb thermometer in large-scale regions such as neighborhoods and cities, numerous equations have been developed to calculate WBGT by using standard meteorological data (Lemke and Kjellstrom 2012). The following equation developed by the Australian Bureau of Meteorology (ABM) was used in this study (ABM 2021):

WBGT = 0.567 T_a_ + 0.393 P_vap_ + 3.94.

The assessment scale of WBGT provides detailed suggestions for outdoor activities with successive temperature ranges (Table S3).

#### PET - physiological equivalent temperature

Physiological Equivalent Temperature (PET) characterizes outdoor conditions by simulating the temperature of a human body with predefined characteristics in thermal equilibrium with the surrounding outdoor air, reflecting conditions typically found in an indoor environment. It is quantified as the outdoor air temperature that would yield the same body temperature as in the reference conditions when the outdoor environment and the reference environment reach thermal equilibrium, utilizing the Munich Energy-Balance Model for Individuals (MEMI) to calculate energy balances. (Höppe 1999). PET assessment scale is derived for different grades of thermal perception of reference human body and shown in Supplemantary Material Table S4

#### UTCI - universal thermal climate index

The Universal Thermal Climate Index (UTCI) operates on the premise that the human body achieves thermal equilibrium with both the ground (via conduction) and the surrounding air (through convection). When the body’s response to thermal stress under actual conditions aligns with that in reference conditions, UTCI corresponds to the ambient air temperature within the reference environment (Blazejczyk et al. 2013; Jendritzky et al. 2012). As part of the COST Action 730 project (Jendritzky et al. 2012), a multi-node model known as UTCI-Fiala was developed. Unlike the Fiala model, UTCI-Fiala does not idealize the human body (as physiological parameters are not fixed), but instead dynamically estimates responses to thermal stress based on real-world reactions through regression methods. Moreover, it accounts for numerous heat transfer mechanisms (Blazejczyk et al. 2010).

There is not a constant value for the thermal resistance of the clothes; it is estimated by the model according to climatic conditions. The stress categories based on UTCI are determined by comparing the physiological responses to actual environmental conditions and to reference conditions (Table S5).

#### PT - perceived temperature

A distinct model that explains the thermal relationship and energy balance of the human body with its environment is the Klima-Michel model, and PT has been developed based on this model. It is described as the air temperature of a reference environment where a human body with a specific set of physiological characteristics perceives heat equally to that of the surrounding environment (Staiger et al. 2012). According to the two-node energy balance model, heat transfer occurs through the skin and respiratory. PT values are assessed with the comfort based thermal perception scale (Table S6).

### Mapping of thermal comfort and statistical analysis

Thermal comfort maps were generated district-based to inform sub-provincial local municipalities about summer heat stress and point to priority areas for action. Based on the observed meteorological parameters and calculated thermal indices, thermal stress levels in the districts were indicated with colors that refer to assessment categories of the indices.

The meteorological observations and thermal comfort data for all districts of İstanbul were analyzed and visualized using RStudio Software. Thermal comfort maps were created with summertime averages of daily mean and daily maximum levels of UTCI, PT, WBGT, AT, PET, and HI. Also, to determine the linear dependence of the meteorological variables and thermal indices, Pearson correlation coefficients (r) were used. Due to the large size of the sample, which was pre-processed and not containing outliers and missing data, Pearson’s correlation analysis was applied, assuming that the data fit a normal distribution based on the Central Limit Theorem. In addition, the stochasticity of the data was increased by random sampling for sultriness and windchill conditions.

## Results

### Spatial variation of meteorological parameters (T_a_, RH, WS and T_mrt_)

**Fig. 2:**
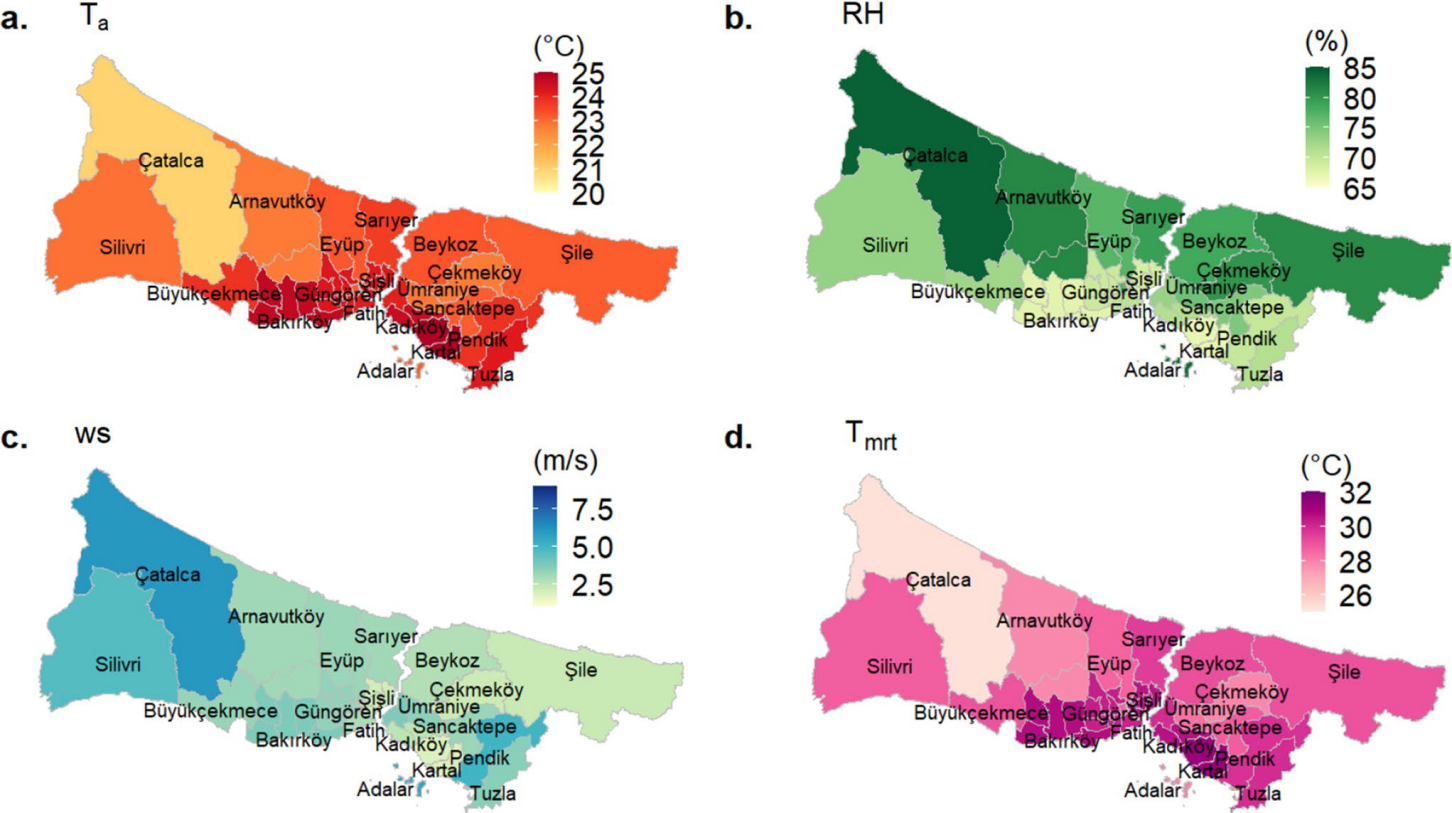
The summer average of T_a_, RH, WS, and T_mrt_ observed in the districts of İstanbul

The summertime (JJA) average air temperatures in all of İstanbul’s distirics range from 20.9 °C to 25.0 °C during 2013–2017 period. The hottest districts are Kartal (25.0 °C), Bakırköy (24.7 °C), and Kadıköy (24.6 °C), respectively, located in the south of İstanbul, where urbanization is intense. The lowest air temperatures for the study period were founded in Çatalca (20.9 °C), Arnavutköy (22.8 °C) on the European side and Çekmeköy (22.7 °C) on the Asian side. The summer-mean temperature in all districts was found 23.9 ± 0.8 °C (Fig. 2a).

The mean relative humidity in İstanbul was found as 72.6 ± 5.1% in summer over the 5-year span. The highest average RH values were observed in Çatalca (84.3%), Arnavutköy (81.4%), and Şile (80.9%) districts, located in the north of İstanbul and having extensive forest lands, and also Prince Island (Adalar) district (83.3%) which is located in the Marmara Sea. The districts where the lowest RH is observed during the summer are Kartal (66.9%), Bakırköy (67.4%), Şişli (68.5%), and neighboring districts with intense urban areas (Fig. 2b).

The summer-mean wind speed measured in İstanbul was 3.2 ± 0.9 m/s and changed in a significant range of 1.8–6.1 m/s over all districts during the study period. The highest values of average wind speed were found in Çatalca (6.1 m/s), Adalar (5.6 m/s), Pendik (5.0 m/s) and Silivri (4.7 m/s) while the lowest values were found in Kartal (1.8 m/s), Şişli (2.1 m/s) and Çekmeköy (2.2 m/s) districts (Fig. 2c).

In İstanbul, the summer-mean T_mrt_ values range from 25.3 °C to 31.2 °C (Fig. 2d) between 2013 and 2017 years and exhibit a similar distribution among districts with respect to the average T_a_ levels (Fig. 2a). The urban heat island effect is strong in the districts of Kartal (with 31.2 °C T_mrt_), Bakırköy (with 30.7 °C T_mrt_), Kadıköy (with 30.6 °C T_mrt_), Şişli (with 30.5 °C), Güngören (with 30.3 °C), and Fatih (with 30.2 °C), which have higher average T_mrt_ values than the average of İstanbul (26.6 ± 1.2 °C). Çatalca (25.3 °C), Arnavutköy (27.8 °C), Çekmeköy (27.9 °C), and Adalar (27.9 °C) are the regions where the lowest T_mrt_ values were found (Fig. 2d).

### Relationships between thermal indices and meteorological variables

The summer average of daily mean and daily maximum values and ranges of heat stress over İstanbul are presented in Table 1. It is noteworthy that there is a difference of about 7 °C to 10 °C between daily average and daily maximum values in PET, UTCI and PT indices.

**Table 1:** Summer thermal comfort levels in İstanbul

	Summer avg. of daily mean levels		Summer avg. of daily maximum levels	
	Mean (°C)	Range (°C)	Mean (°C)	Range (°C)
AT	24.6 ± 1.2 °C	19.6–26.6	28.1 ± 1.3 °C	23.5–30.8
HI	24.5 ± 1.2 °C	20.5–26.1	29.2 ± 1.0 °C	25.6–31.0
WBGT	25.8 ± 0.5 °C	24.0-26.4	28.1 ± 0.6 °C	26.5–29.4
PET	19.6 ± 1.4 °C	14.5–21.7	29.2 ± 1.7 °C	23.3–31.9
UTCI	19.8 ± 1.9 °C	11.5–22.5	27.8 ± 1.4 °C	22.9–30.0
PT	18.2 ± 1.1 °C	14.0–20.0	26.4 ± 1.4 °C	21.4–29.0

When it is focused on how meteorological variables affect the thermal comfort, the most critical meteorological parameter for simple indices has been the air temperature at all districts. As indicated in Table 2, very significant correlations were observed between T_a_ and AT, HI and WBGT indices with mean correlation coefficients of 0.90, 0.90, and 0.87, respectively. For PET, UTCI and PT, T_mrt_ has also had great importance, along with T_a_. The mean correlation coefficients of the districts detected between T_mrt_ and PET, UTCI, and PT indices were 0.93, 0.87, and 0.88, respectively.

**Table 2:** The mean, minimum and maximum correlation coefficients between meteorological variables (T_a_, RH, WS, and T_mrt_) and thermal indices (AT, HI, WBGT, PET, UTCI, and PT) at all districts of İstanbul

		T_a_ (°C)	RH (%)	WS (m/s)	T_mrt_ (°C)
AT (°C)	mean r ± s.d. min.-max.	0,90 ± 0,05 0,73 − 0,96	-0,31 ± 0,14 (-0,16)-(-0,50)	0,16 ± 0,12 0,01 − 0,37	0,55 ± 0,11 0,14 − 0,70
HI (°C)	mean r ± s.d. min.-max.	0,90 ± 0,04 0,73 − 0,93	-0,74 ± 0,07 (-0,61)-(-0,85)	0,44 ± 0,11 0,15 − 0,62	0,73 ± 0,07 0,51 − 0,83
WBGT (°C)	mean r ± s.d. min.-max.	0,87 ± 0,03 0,84 − 0,93	-0,20 ± 0,13 0,01-(-0,41)	0,29 ± 0,09 0,12 − 0,49	0,53 ± 0,10 0,20 − 0,65
PET (°C)	meanr ± s.d. min.-max.	0,90 ± 0,04 0,72 − 0,93	-0,67 ± 0,08 (-0,42)-(-0,80)	0,33 ± 0,13 0,04 − 0,55	0,93 ± 0,03 0,85 − 0,96
UTCI (°C)	meanr ± s.d. min.-max.	0,88 ± 0,05 0,67 − 0,92	-0,55 ± 0,10 (-0,23)-(-0,69)	0,22 ± 0,11 0,04 − 0,42	0,87 ± 0,04 0,73 − 0,92
PT (°C)	meanr ± s.d. min.-max.	0,91 ± 0,03 0,79 − 0,93	-0,58 ± 0,09 (-0,34)-(-0,73)	0,35 ± 0,13 0,11 − 0,56	0,88 ± 0,03 0,78 − 0,92

The maximum correlation coefficients between T_a_ and thermal indices were seen in Şile for AT (0.96) and WBGT (0.93), in Kadıköy for HI (0.93), in Eyüp for PET (0.93), and in Çekmeköy for UTCI (0.92) and PT (0.93). The minimum correlation coefficients between T_a_ and thermal indices were determined in Adalar for AT (0.73), PET (0.72), UTCI (0.67) and PT (0.79), in Çatalca for HI (0.73), and in Üsküdar for WBGT (0.84).

While relative humidity and thermal indices have negative correlations in all districts, between wind speeds and thermal indices, a weak and positive relationship has been detected in all districts except Çatalca and Adalar. However, this was found to be counterintuitive, because in the case of overheating generally in summer months at high temperatures with rising humidity the sensible temperature also increases, whereas at relatively low temperatures the “heat stress” is reduced by the wind. To examine the sultriness effect of the relative humidity at high temperatures and the windchill effect at low temperatures, all meteorological and thermal indices data were rearranged. The sultriness and windchill cases were defined respecting Steadman’s original tables (Steadman 1979a, b) and the conditions were listed in Table S7

**Fig. 3:**
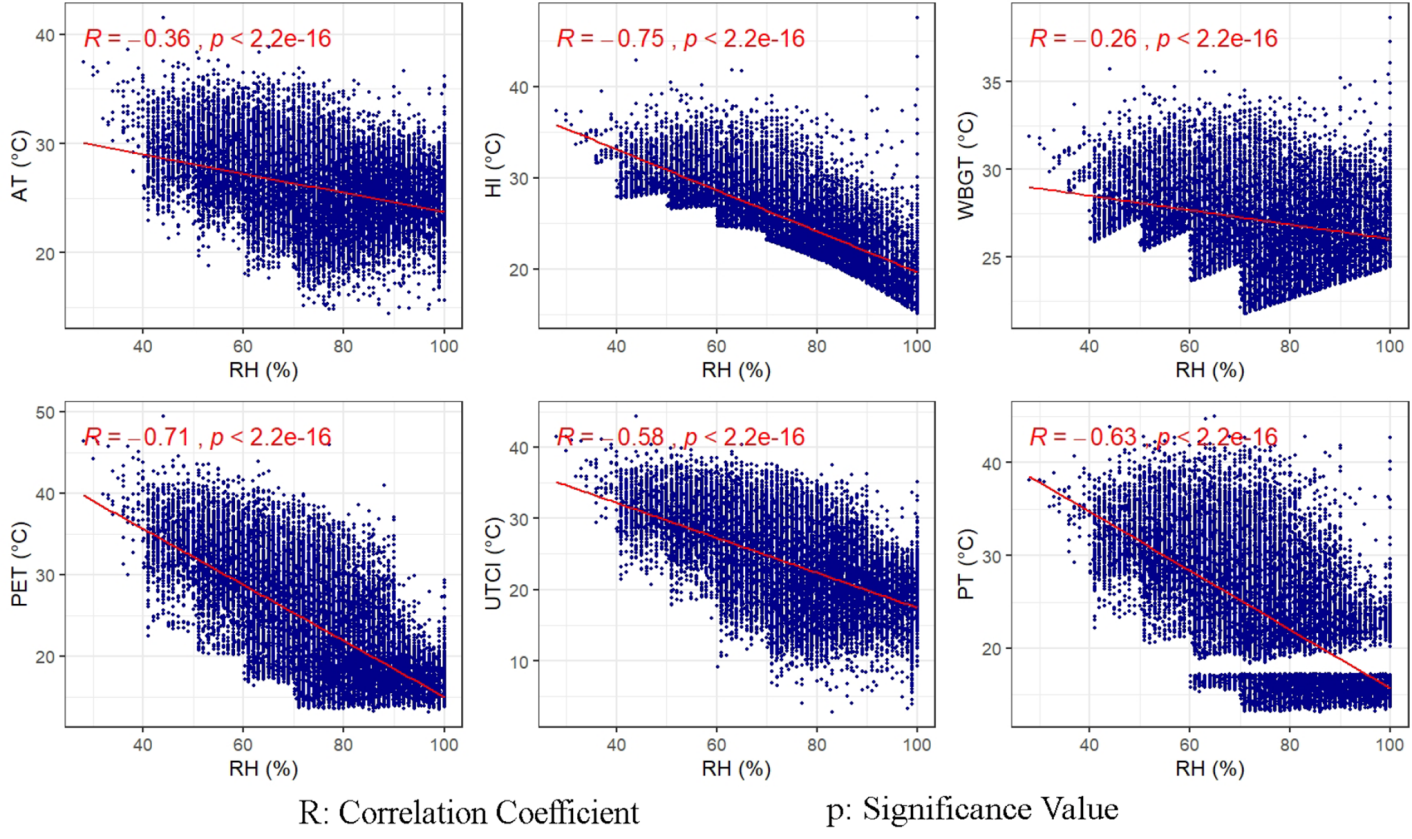
The relationships between relative humidity and thermal indices in the sultriness case

**Fig. 4:**
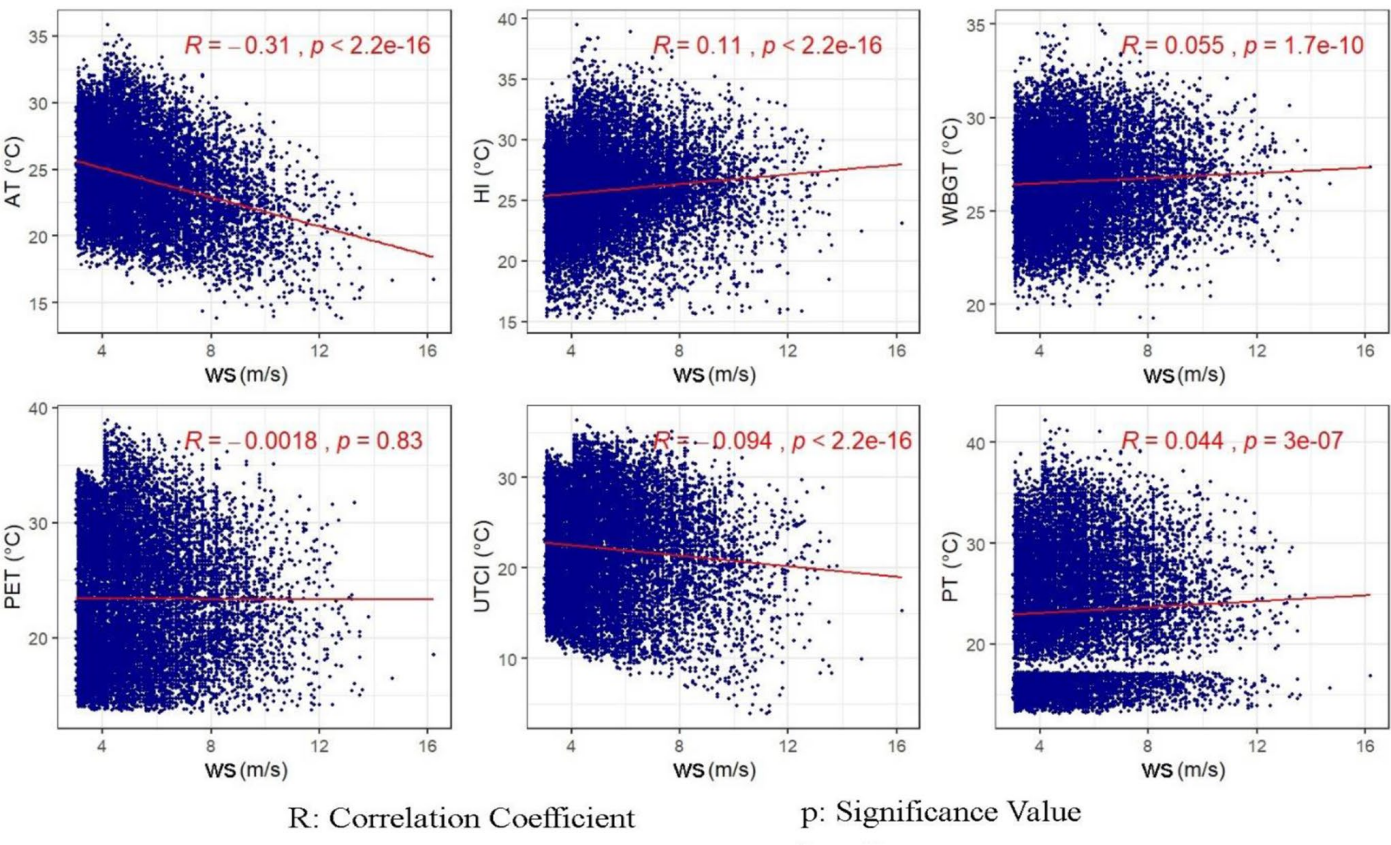
The relationship between wind speed and thermal indices in the wind chill case

The new datasets of the sultriness and windchill cases were created, and the data size was reduced to analyze by random sampling of 10% of the dataset. The associations of thermal indices with RH and WS in the cases of sultriness and windchill effect were analyzed by scatterplots and correlation coefficients in Figs. 3 and 4, respectively.

In the sultriness case, RH still exhibits negative relationships with all thermal indices (Fig. 3). As to windchill case, the directions of the correlations between WS-AT, WS-UTCI, and WS-PET have turned negative, but the strength of all correlations has been weak or none as indicated in Fig. 4. The maximum correlation was found as 0.31 between WS and AT indices.

Since relative humidity is the ratio of the amount of water vapor in a specific volume of air to the maximum amount of water vapor in that volume, it may not be considered as an indicator of the actual moisture content in the air. Therefore, in order to see the effect of humidity on thermal stress, the correlations between vapor pressures and thermal indices were also examined. However, since the measured vapor pressure values contain large amounts of missing data, vapor pressures calculated with relative humidity values were used in the analyses. The scatterplots and correlation coefficients between vapor pressure and thermal indices are given in the Supplementary Material, Figure S2

In contrast to the negative correlations observed between thermal indicators and relative humidity, the direction of the correlations established with vapor pressure was seen to be positive (Figure S2). This suggests that on days with higher vapor pressure (indicating more moisture in the air), the thermal indices tend to be higher, implying greater heat stress. This relationship is particularly strong with the WBGT index, as shown by the high correlation coefficient (*R* = 0.76), which is a measure specifically designed to incorporate humidity and is used to estimate heat stress in direct sunlight.

### Diurnal variations of the meteorological variables and thermal indices

**Fig. 5:**
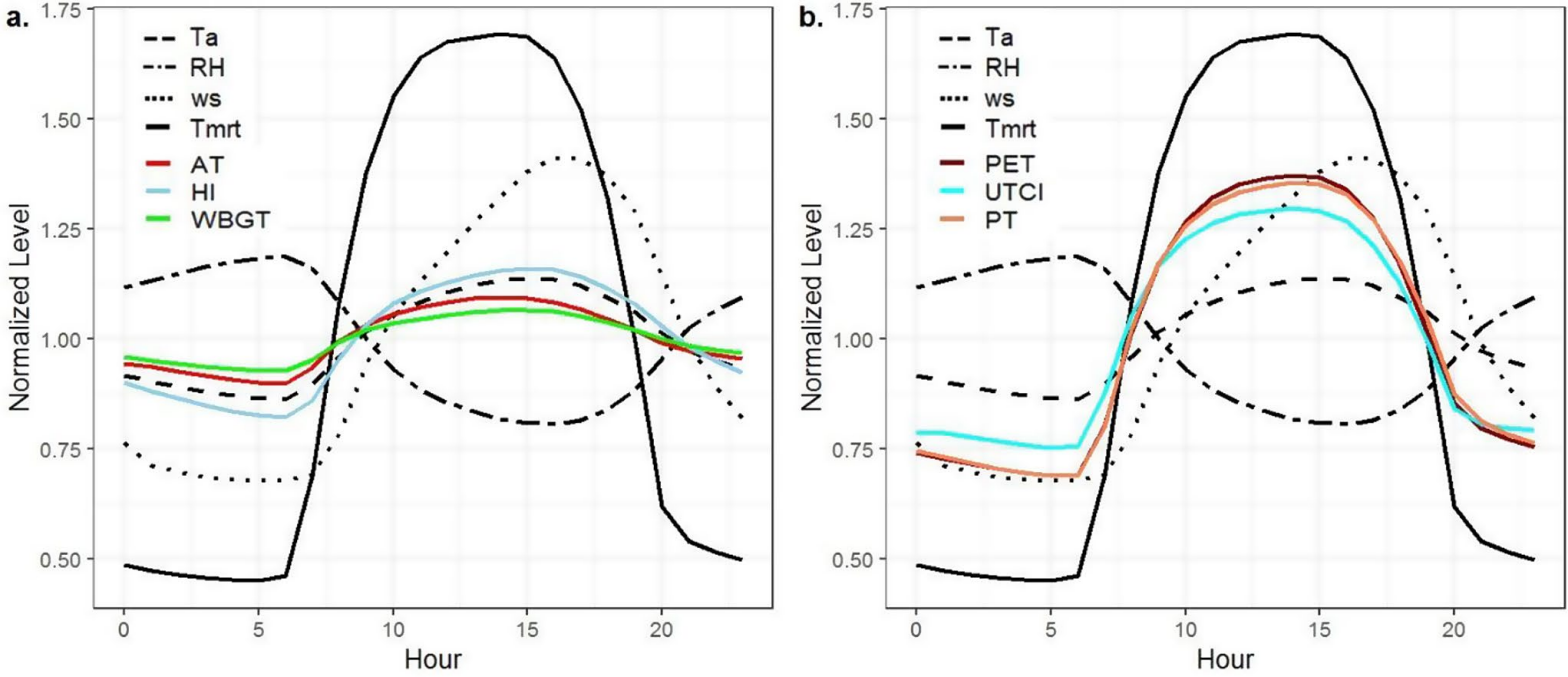
Diurnal cycles of meteorological parameters (T_a_, RH, WS, T_mrt_) and **(a)** simple indices (AT, HI, WBGT), **(b)** energy balance-based indices (PET, UTCI, PT)

Figure 5 shows the normalized data along with the all hourly meteorological parameters and thermal indices. It was noted that the air temperature began to rise at sunrise (at 06:00 LST), peaked (26.9 °C) in the afternoon (at 15:00 LST), and then began to fall during the night. Although the incoming energy from the sun is at its maximum level at noon (around 12:00–13:00 LST), the air temperature reaches its maximum value in the afternoon, as the earth-atmosphere system continues to gain energy from the terrestrial radiation.

As air temperature increases, the air’s ability to hold moisture also rises. Consequently, relative humidity tends to decrease while T_a_ increases during daylight hours. Meanwhile, the T_mrt_ serves as an equivalent surface temperature, reflecting the combined impact of all shortwave and longwave radiation that the human body encounters. During daytime hours, T_mrt_ shows a more pronounced increase than T_a_, primarily driven by the influence of shortwave radiation. The peak value for T_mrt_, with an average of 49.5 °C, occurs around midday (12:00–15:00 LST) due to the intense solar radiation.

While the daily variations of AT, HI and WBGT were similar to T_a_ during the day (Fig. 5a), PET, UTCI and PT indices showed similar variations to both T_mrt_ and T_a_ parameters (Fig. 5b). Although HI and WBGT reached their maximum levels (mean of 28.1 °C and 27.5 °C respectively) at 15:00 LST, the highest values of AT (26.6 °C), PET (30.2 °C), UTCI (28.4 °C), and PT (29.3 °C) were observed at 14:00 similar to T_mrt_. In the daytime, Energy balance-based indices (PT, UTCI, and PET) have been observed higher than T_a_ with the influence of T_mrt_ variable.

### Regional thermal comfort analysis

**Fig. 6:**
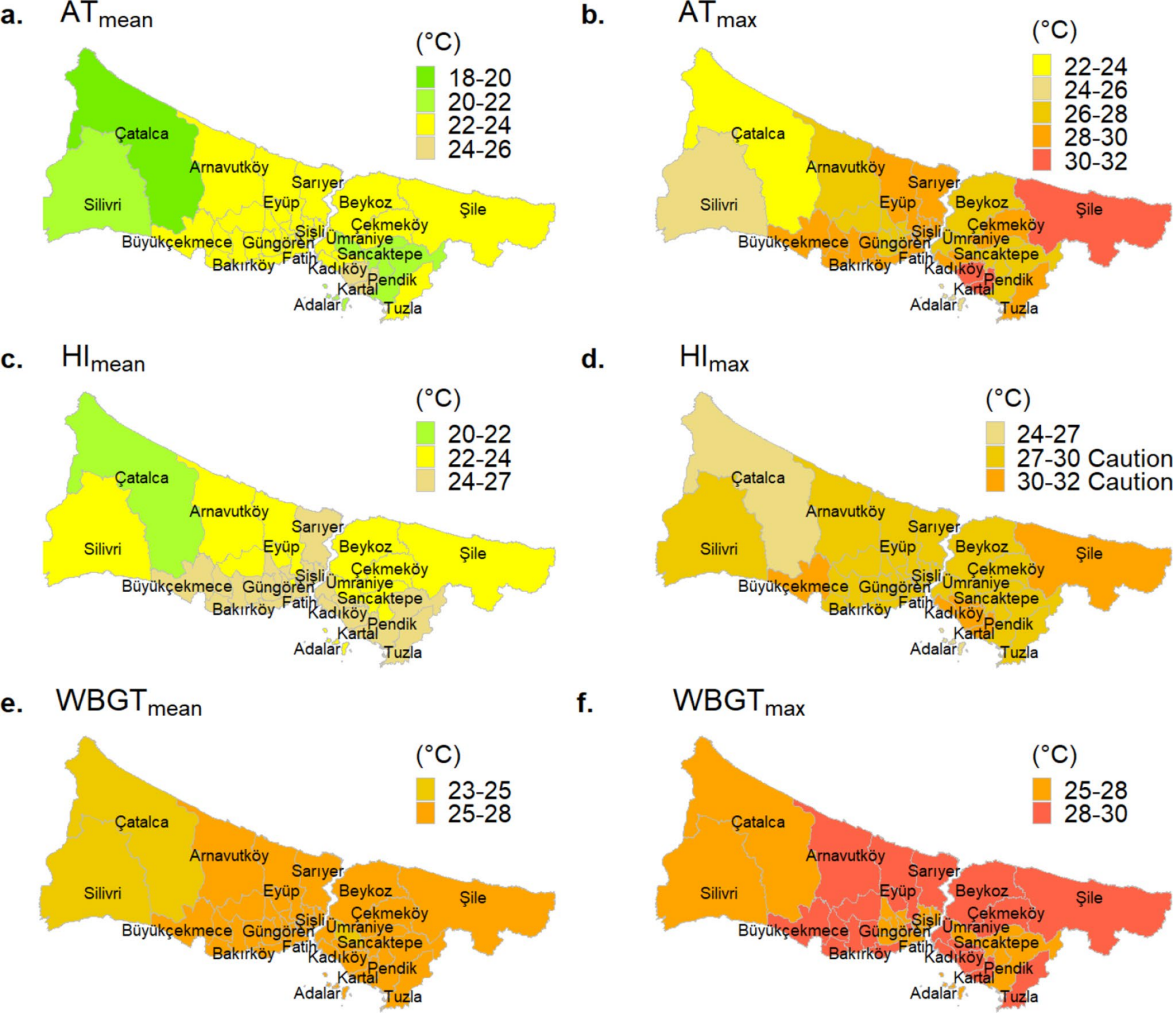
The variation of summer average of daily mean and daily maximum AT, HI, and WBGT levels in İstanbul

In this study, the daily mean and daily maximum thermal indices were evaluated. The summer averages of the daily mean and maximum thermal stress levels observed all over İstanbul districts with the AT, HI, and WBGT indices are shown in Fig. 6. According to Fig. 6a, the summer average of AT_mean_ levels is between 22 and 24 °C in most districts of İstanbul. This value is getting below 22 °C in Pendik, Sultanbeyli, Sancaktepe, Ümraniye, Silivri, and Çatalca districts, where the wind speed is high because AT index takes into account wind speed measurements and windchill effect on thermal comfort. In comparison to the daily mean and maximum of HI and WBGT indices, AT_max_ values have a wide range and indicate spatial variation discriminately (Fig. 6b).

As shown in Fig. 6c, the HI_mean_ levels (Table 1) do not present a risk for human health in İstanbul during the summer. However, HI_max_ levels give a warning with prolonged exposure to those levels due to thermal stress in all districts with the exception of Çatalca (Fig. 6d). Although the 27–32 °C range gets involved in the *Caution* category in the HI assessment table (Table S2), we split the category ranges into sub-categories and colorized them differently, similar to all thermal indices maps, to see the micro-level differences of districts.

WBGT_mean_ values varied between districts from 24.0 °C to 26.4 °C, and WBGT_max_ from 26.5 °C to 29.4 °C. A significant regional difference could not be detected due to the small ranges of the summer average of the WBGT indices (Fig. 6e and f). According to WBGT levels, people in all districts have been exposed to thermal stress in the summer months and should pay attention to the recommendations for outdoor activities in the WBGT assessment table (Table S3).

**Fig. 7:**
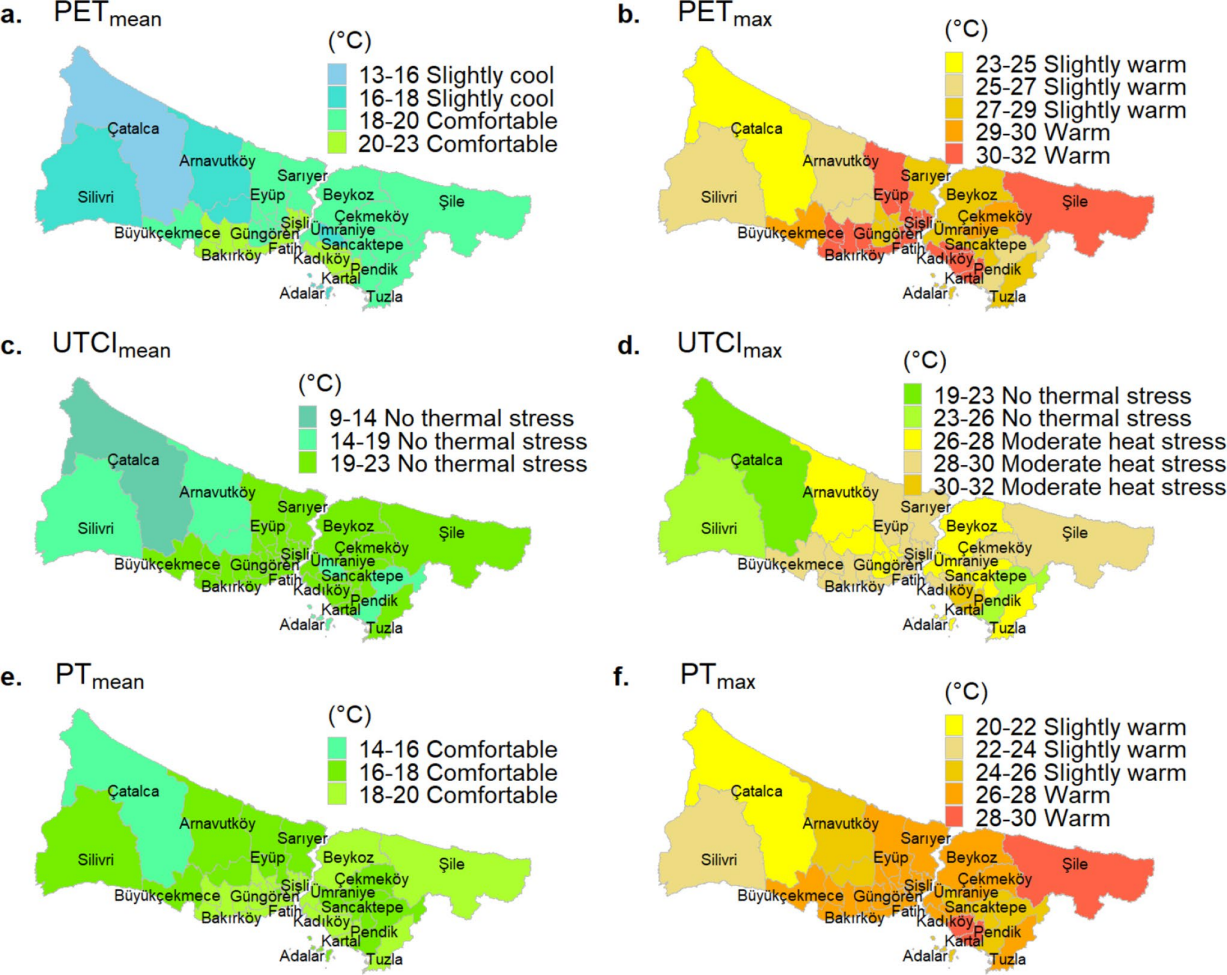
The variation of summer-mean and summer-mean daily maximum PET, UTCI, and PT levels in İstanbul

Figure 7 displays summer thermal comfort maps generated using daily mean and daily maximum PET, UTCI, and PT indices. It has been observed that there is a remarkable difference between the daily maximum and daily mean thermal stress levels. Because the energy balance-based indices have a very strong association with the T_mrt_ parameter, which has changed over a wide range during the day due to the shortwave radiation from the sun (Fig. 5).

As seen in Fig. 7a and c, and 7e, the daily mean PET, UTCI, and PT levels do not support the heat stress throughout the city. Even though it is summer, slightly cool conditions based on the PET index are reported in some parts of the city (Çatalca, Adalar, Silivri, Arnavutköy, Başakşehir, and Ümraniye).

PET_max_ and PT_max_ levels, however, indicate that thermal comfort conditions could not be maintained during the day in all districts, and slight to moderate heat stress was noted (Fig. 7b and f). The thermal stress levels in Sarıyer, Beykoz, Üsküdar, Tuzla, Güngören, and Esenler throughout the summer have been reported as slight heat stress with PET_max_ and moderate heat stress with PT_max_. According to the UTCI_max_ map, slight to moderate heat stress was experienced by all districts in İstanbul except Çatalca, Silivri, and Pendik (Fig. 7d).

For the reason the reference environment conditions and the human characteristics assumed in the definition of indices are different, the heat stress levels and assessment ranges in the districts vary with thermal indices.

## Discussion

Spatial variations in summer thermal comfort are complex and influenced by a variety of factors, including climate, geography, and the built environment. In this work the southern part of İstanbul, consisting of Adalar, Bakırköy, Büyükçekmece, Fatih, Kadıköy, Kartal, Silivri, and Tuzla districts, has indicated different meteorological characteristics than the northern part, which includes Arnavutköy, Çatalca, Sarıyer, Beykoz, and Şile. The summer average of air temperatures and mean radiant temperatures were higher in the south of İstanbul, in districts with dense urban settlements, than in the northern regions; while relative humidity is higher in northern districts with forests and green areas, as in the study of Cheung et al. (2021). However, the wind speed measurements have varied in a different regional pattern in comparison with T_a_, T_mrt_, and RH. This may be explained by the complex topography and land-sea breeze effect over the coastal parts of the city (Deniz et al. 2011; Incecik 1996; Ünal et al. 2020).

The outdoor thermal indices provide a more accurate measure of human’s welfare and physiological response to thermal stress than meteorological variables. Relationships between thermal indices and meteorological variables have been investigated in order to determine their applicability as an alternative to meteorological variables in defining regional climate characteristics. T_a_ and T_mrt_ have very strong and positive correlations with all thermal indices. Despite this strong relationship, when the measured air temperature and calculated thermal stress maps are analyzed together, it is seen that the regional patterns do not completely overlap. Therefore, it is concluded that it would not be correct to make inferences related to the problems that may be caused by thermal comfort and heat stress by just looking at the measured air temperatures, and it is important to choose the most appropriate thermal index for the specific region.

It is well known that in hot weather the moisture content of the air rises with increasing evaporation and this produces a sultriness effect that increases thermal stress. Positive relationships between vapor pressure and thermal indices confirm this phenomenon. However, when examining directly the effect of measured relative humidity on thermal stress, negative correlations were observed between relative humidity and thermal indices in all districts, similar to the findings of previous studies (Blazejczyk et al. 2012; Zare et al. 2018). It has been determined that the sultriness effect of RH cannot be explained with a linear model since the diurnal relative humidity varies in an inverse cycle with the air temperature and thermal indices.

Similarly, windchill effects on thermal indices could not be inferred clearly from correlation coefficients. While the general expectation is that increased wind speed should reduce heat stress caused by overheating, the observed positive correlation in the study could be influenced by specific local climatic conditions, urban microclimate effects, and relative humidity levels. In urban settings like İstanbul, building density, urban geometry, and surface materials can influence local wind patterns and thermal comfort. For instance, in densely built areas, wind might not effectively reduce heat stress due to limited airflow or the presence of heat-absorbing materials. The interplay between wind speed and relative humidity can also affect thermal comfort. In high humidity conditions, evaporation is less effective, and increased wind speed might not provide the usual cooling effect.

In all the thermal comfort maps, a significant difference was seen between the daily mean and daily maximum values of the thermal indices due to the variations in insolation and air temperature between day and night. In particular, in PET, UTCI, and PT, which are energy balance-based indices, the difference has increased because of the mean radiant temperature (T_mrt_) parameter. Also, the PET and PT indices were determined to be more appropriate than the UTCI for the regional thermal comfort analysis in İstanbul because they clearly showed the differences in thermal perception levels between the districts. These comparisons are important to decision-making on appropriate thermal indices for regional thermal comfort analysis.

The districts with the most intense heat stress are Kartal and Maltepe, according to the AT, HI, PET, and UTCI indices, and Şile according to the WBGT and PT indices. The highest average air temperature, the lowest average relative humidity, and the lowest average wind speed are all seen in Kartal during the summer. All indices indicate that thermal stress is at its lowest in Çatalca, where the measured summer mean air temperature is the lowest and the summer average relative humidity and wind speeds are the highest.

It is also observed that not only meteorological characteristics but also the type of land use in districts have an impact on regional thermal comfort, similar to the findings in the previous studies (Cohen et al. 2013; Sodoudi et al. 2018; Zeren Cetin and Sevik 2020). Urban areas are often covered by concrete and asphalt, which absorb and retain heat, whereas forested areas provide shade and help cool the environment through transpiration. While Çatalca, which is surrounded by forested areas, is low in terms of all thermal indices, urbanized districts like Fatih, Şişli and Kadıköy, where there are more settlements and buildings, are higher.

The PET index was frequently used in studies conducted in Europe to determine urban heat stress levels. Compared to other big cities in the Mediterranean Region, which is one of the most vulnerable regions against heatwaves, it has been observed that calculated PET values during summer months in İstanbul remained at low levels in most regions. In the comparison of İstanbul’s thermal stress in summer with other cities, it was ensured that the reference studies included the hottest period observed in those cities and that the same thermal index (PET) was used in the evaluations. Within the Mediterranean Region, both daily mean and daily maximum PET values were taken into account in order to cover as many locations as possible in this assessment. Summer-average of daily mean PET levels have ranged from 14.5 °C to 21.7 °C, and the average of the districts was found 19.6 °C in İstanbul, while at the urban centers of Seville and Madrid, daily mean PET values in the hottest months were observed 37.8 °C and 32.9 °C, respectively (Karimi and Mohammad 2005). According to the study of Cohen et al. (2013) conducted in Tel Aviv, daily maximum PET levels are recorded in average, as 24.9 °C in the parks, 40.0 °C in the squares and 43.6 °C in the streets over the summer months. Whereas in İstanbul, summer-average of daily maximum PET levels change from 23.3 °C to 31.9 °C regionally, and the average of the districts is recorded as 29.2 °C.

According to the standard assessment ranges (Matzarakis et al., 1999) given in Table S.4, it can be stated that thermal comfort can be provided in İstanbul with summer-average of daily mean PET values (19.6 °C) staying within the “comfortable/neutral” sensation range (18–23 °C); the average of daily maximums (29.2 °C) corresponds to the “warm” category (29–35 °C). However, due to the adaptation of people living in different climatic regions, the thermal comfort range may vary depending on the region and heat stresses may be perceived at more acceptable levels in comparison to the standard ranges. In order to set region-specific thermal comfort ranges, the degree of satisfaction is usually determined by questioning people with a survey method and the standard calculated PET values are modified by relating them to the survey results (Lin and Matzarakis 2008; Salata et al. 2016; Tseliou et al. 2017). Using this method, Karimi and Mohammad (2005) determined the “comfortable” PET ranges for Seville and Madrid squares as 28.42–30.87 °C and 24.5–29.82 °C, respectively; Cohen et al. (2013) noted that “no thermal stress” was felt in Tel Aviv when the observed PET values were between 19 and 26 °C. In Athens, the thermally acceptable PET range was found to be 26.0–32.0 °C (Tseliou and Tsiros 2016), while Potchter et al. (2018), who evaluated multiple cities together, accepted a modified “thermal comfort” range (PET) of 24–26 °C in hot climates including the Mediterranean region. Unfortunately, no such study has yet been conducted to identify the thermal comfort levels for İstanbul; therefore, the thermal stress categories were determined according to the standard assessment ranges.

The approach of using relative humidity is another limitation of this study due to the lack of regular vapor pressure measurement data. It is also admitted that during the comparison of different thermal indicators, relative humidity might not be able to precisely reveal the moisture content of the air or the related heat stress, and this could be a limitation of the study. However, since the regional thermal comfort assessments are based on the heat stress levels and variability observed in the districts, the weak correlations between meteorological variables and thermal indices can be ignored.

## Conclusion

In this study, regional thermal stress levels were associated with topographic and meteorological characteristics, and the effectiveness of different thermal indices was investigated to define sub-climatic characteristics. The main consequences of the study are specified as below:

- The stress categories of thermal indices observed in districts and the variation of thermal comfort have differed from each other due to the different assumptions and algorithms for defining thermal indices.
- Thermal perception differences between districts were seen more clearly in the thermal comfort maps created with the AT, PET, and PT indices. HI, WBGT, and UTCI maps were found insufficient for the regional analysis of thermal comfort or thermal stress in İstanbul, because of the inability to observe micro-level differences among the districts.
- As daily maximum values provide a more realistic representation of increased thermal stress throughout the day, these indicators are expected to be more effective than daily average values in determining health risks associated with temperature/thermal comfort.
- Although the thermal indices have had very strong correlations with T_a_ and T_mrt_, the thermal stress/comfort maps determined with all indices have shown different distributions from T_a_ and T_mrt_ maps.
- Due to its location and climatic features, the daily mean and maximum PET values were lower compared to other cities in the Mediterranean Region. However, since there is not an assessment scale specific to İstanbul, it was not possible to make a comparison on how thermal stress is perceived.

The thermal indices and thermal comfort maps might be helpful in future studies, in the direction of the United Nations’ sustainable development goals. Thermal indices can be used to better understand how climate change affects thermal conditions, and to held local governments decide what mitigation and adaptation measures to take (SDG 13 – Climate Action). Also, it can be used to analyze to heat-related health impacts to organize public health interventions (SDG 3 – Good Health and Well-being). Finally, thermal comfort maps can be used to identify urban heat islands, to create efficient strategies for enhancing human well-being in hot weather, and to make decisions about urban planning and design (SDG 11 – Sustainable Cities and Communities).

### Electronic supplementary material

Below is the link to the electronic supplementary material.

Supplementary Material 1

## Data Availability

We have obtained the meteorological data from the Turkish State Meteorological Service (TSMS), but not allowed to share the data. Therefore data cannot be made available. **Author Contribution Statement**: **M. Y.**: Conceptualization, Project administration, Investigation, Original draft writing, Review & editing, Visualization, Statistical analysis. **Y.K.**: Visualization, Statistical analysis, Review & editing. **H.T.**: Supervision, Review & editing. **S.İ.**: Conceptualization, Investigation, Review & editing.
