# Supplementary material for: Analysis of the summer thermal comfort indices in İstanbul
Source: Int J Biometeorol. 2024 Apr 24;68(7):1327–42. doi: 10.1007/s00484-024-02669-7 (PMC11272817; doi:10.1007/s00484-024-02669-7)
Supplement: Supplementary file 1 — Supplementary Material 1 [file 484_2024_2669_MOESM1_ESM.docx]

# Analysis of Spatial Variability of the Summer Thermal Comfort Indices in İstanbul – Supplementary Material

**Table S.1.** Specifications of TSMS stations observing meteorological variables in İstanbul between 2013-2017 and missing value rates

|  | **Stations/District** | **Coordinates** | **Altitude (m)** | **Climatic features** | **Corine Land Cover Type** | **Observed variables and % of missing values** |
| --- | --- | --- | --- | --- | --- | --- |
| **1** | Adalar (Buoy)/ Adalar | 40.9328  28.9489 | 0 | South, coastal | Sea and ocean | T_a_ (39%)  RH (43%)  WS (38%) |
| **2** | Arnavutköy/ Arnavutköy | 41.2203  28.7075 | 140 | Inland, rural | Discontunious Urban Fabric | T_a_ (26%)  RH (29%)  WS (26%) |
| **3** | Florya/Bakırköy | 40.9758  28.7865 | 37 | South, coastal, urban | Green Urban Areas | T_a_ (2%)  RH (9%)  WS (1%) |
| **4** | Atatürk Havalimanı/ Bakırköy | 40.9819  28.8208 | 33 | South, coastal, urban | Airports | T_a_ (2%)  RH (2%)  WS (4%)  C (2%) |
| **5** | Beykoz/Beykoz | 41.1417  29.0739 | 5 | Bosphorus,coastal, urban | Industrial or Commercial Units | T_a_ (24%)  RH (25%)  WS (26%) |
| **6** | Beykoz Anadolu Feneri/Beykoz | 41.2233  29.1658 | 58 | North, coastal, forest | Broad Leaved Forest | T_a_ (33%)  RH (33%)  WS (32%) |
| **7** | Büyükçekmece/ Büyükçekmece | 41.0453  28.5900 | 20 | South, coastal, urban | Industrial or Commercial Units | T_a_ (2%)  RH (7%)  WS (3%) |
| **8** | Çatalca Radar Sahası/Çatalca | 41.3409  28.3568 | 381 | Inland, forest | Broad Leaved Forest | T_a_ (3%)  RH (19%)  WS (3%) |
| **9** | Çekmeköy/ Çekmeköy | 41.0783  29.3256 | 80 | Inland, forest | Discontunious Urban Fabric | T_a_ (23%)  RH (29%)  WS (25%) |
| **10** | Eyüp/Eyüp | 41.1028  28.9242 | 54 | Inland, urban | Pastures | T_a_ (3%)  RH (4%)  WS (3%) |
| **11** | Kumkapı Fener/ Fatih | 41.0042  28.9547 | 12 | South, coastal, urban | Discontunious Urban Fabric | T_a_ (21%)  RH (23%)  WS (25%) |
| **12** | Fatih/Fatih | 41.0155  28.9601 | 10 | Inland, urban | Discontunious Urban Fabric | T_a_ (1%)  RH (8%)  WS (1%) |
| **13** | Davutpaşa Marmara/ Güngören | 41.0266  28.8853 | 68 | Inland, urban | Sport and Leisure Facilities | T_a_ (23%)  RH (25%)  WS (21%) |
| **14** | Göztepe/Kadıköy | 40.9890  29.0532 | 41 | South, inland, urban | Industrial or Commercial Units | T_a_ (35%)  RH (35%)  WS (33%) |
| **15** | Kadıköy Rıhtım/ Kadıköy | 40.9883  29.0190 | 5 | South, coastal, urban | Green Urban Areas | T_a_ (3%)  RH (4%)  WS (2%) |

|  | **Stations/District** | **Coordinates** | **Altitude (m)** | **Climatic features** | **Corine Land Cover Type** | **Observed variables and % of missing values** |
| --- | --- | --- | --- | --- | --- | --- |
| **16** | İstanbul Bölge/ Kartal | 40.9113  29.1558 | 18 | South, coastal, urban | Industrial or Commercial Units | T_a_ (0.4%)  RH (1%)  WS (2%)  C (23%) |
| **17** | Sabiha Gökçen Havalimanı/ Pendik | 40.8977  29.3033 | 99 | Inland, urban | Airports | T_a_ (0.8%)  RH (0.8%)  WS (2%)  C (0.5%) |
| **18** | Sancaktepe/ Sancaktepe | 41.0086  29.2822 | 110 | Inland, urban | Industrial or Commercial Units | T_a_ (23%)  RH (30%)  WS (24%) |
| **19** | Samandıra Havalimanı/ Sancaktepe | 40.9866  29.2135 | 123 | Inland, urban | Airports | T_a_ (4%)  RH (7%)  WS (3%) |
| **20** | Sarıyer/Sarıyer | 41.1464  29.0502 | 59 | Bosphorus,coastal, urban | Green Urban Areas | T_a_ (0.1%)  RH (1%)  WS (0.2%)  C (18%) |
| **21** | Kumköy-Kilyos/ Sarıyer | 41.2505  29.0384 | 38 | North, coastal, forest | Discontunious Urban Fabric | T_a_ (0.4%)  RH (0.9%)  WS (0.8%)  C (42%) |
| **22** | Silivri/Silivri | 41.1856  28.1575 | 195 | Inland, rural | Broad Leaved Forest | T_a_ (27%)  RH (28%)  WS (28%) |
| **23** | Silivri Fener/ Silivri | 41.0731  28.2394 | 9 | South, coastal, sub-urban | Port Areas | T_a_ (9%)  RH (12%)  WS (8%) |
| **24** | Şile/Şile | 41.1688  29.6007 | 83 | North, coastal, sub-urban | Discontunious Urban Fabric | T_a_ (2%)  RH (3%)  WS (2%) |
| **25** | Şişli/Şişli | 41.0547  28.9683 | 60 | Inland, urban | Road and Rail Networks and Associated Land | T_a_ (28%)  RH (28%)  WS (29%) |
| **26** | Tuzla İTÜ/Tuzla | 40.8128  29.2978 | 13 | South, coastal, urban | Port Areas | T_a_ (7%)  RH (10%)  WS (7%) |
| **27** | Tuzla Aydınlı/ Tuzla | 40.8514  29.2717 | 8 | South, coastal, urban | Port Areas | T_a_ (6%)  RH (9%)  WS (6%) |
| **28** | Tuzla/Tuzla | 40.8278  29.2931 | 3 | South, coastal, urban | Industrial or Commercial Units | T_a_ (2%)  RH (2%)  WS (3%) |
| **29** | Ümraniye/ Ümraniye | 41.0294  29.1383 | 191 | Inland, urban | Coniferous Forest | T_a_ (26%)  RH (26%)  WS (26%) |
| **30** | Üsküdar/Üsküdar | 41.0328  29.0464 | 75 | Bosphorus,coastal, urban | Discontunious Urban Fabric | T_a_ (28%)  RH (27%)  WS (26%) |


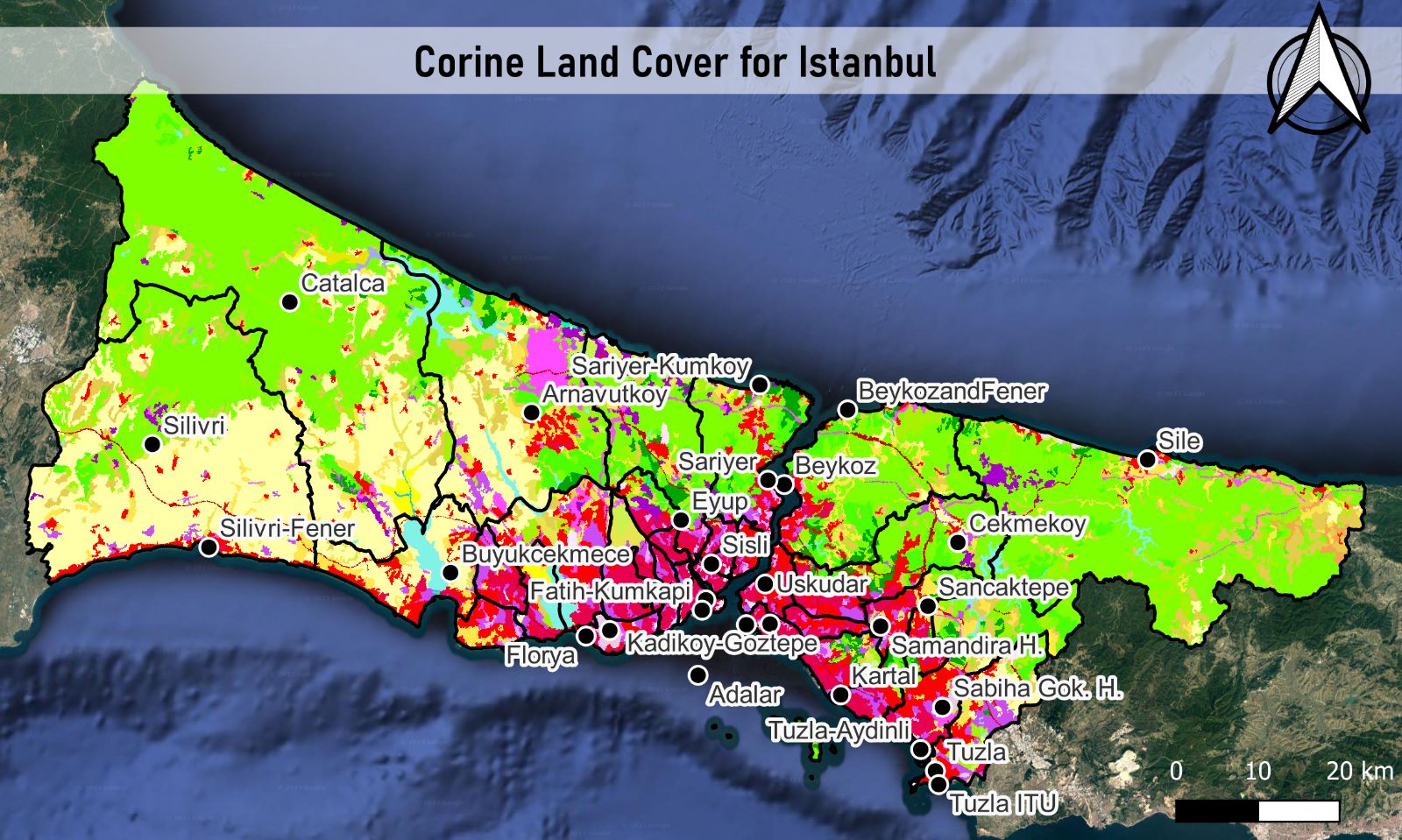


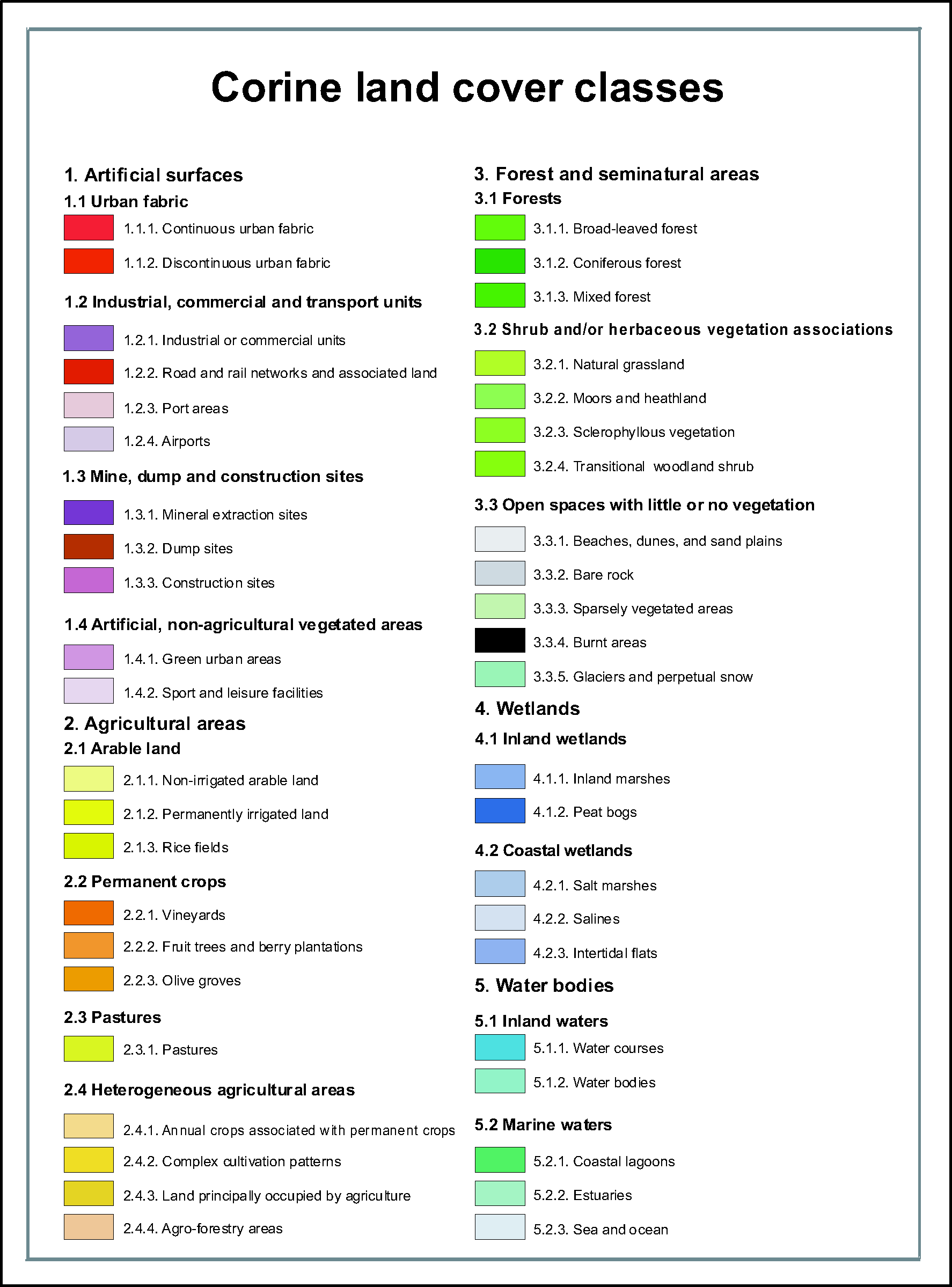


**Figure S.1.** CORINE Land Cover for İstanbul

**AT Calculation Approaches by Steadman (1994)**

- Temperature and vapor pressure dependent approach:

AT_p_ = 0,89 T + 0,38 P_vap_ – 2,56

AT_p_ : Apparent temperature (°C), T : Temperature (°C), P_vap_ : Vapor pressure (hPa)

- Temperature, vapor pressure and wind speed dependent approach:

AT_pv_ = T + 0,33 P_vap_ – 0,7 ν_10_ – 4,00

AT_pv_ : Apparent temperature (°C), ν_10_ : Wind speed measured at 10 m height (m/s)

- Temperature, vapor pressure, wind speed and radiation dependent approach:

AT_pvg_ = T + 0,35 P_vap_ – 0,7 ν_10_ + 0,7 Q_g_ / (ν_10_ + 10) – 4,25

AT_pvg_ : Apparent temperature (°C), Q_g_ : Absorbed radiation per unit body surface (W/m^2^)

**Assessment Tables of Thermal Indexes**

**Table S.2.** Assesment table of HI (NWS, 2021)

| **HI (°C)** | **Category** | **Possible heat disorders for people in high risk groups** |
| --- | --- | --- |
| 27-32 | Caution | Fatigue possible with prolonged exposure and/or physical activity. |
| 33-41 | Extreme Caution | Sunstroke, muscle cramps, and/or heat exhaustion possible with prolonged exposure and/or physical activity. |
| 42-54 | Danger | Sunstroke, muscle cramps, and/or heat exhaustion likely. Heatstroke possible with prolonged exposure and/or physical activity. |
| >54 | Extreme Danger | Heat stroke or sunstroke likely. |

**Table S.3.** Assessment table of WBGT (ABM, 2021b)

| **WBGT (°C)** | **Recommended outdoor activities** |
| --- | --- |
| <18 | Unlimited |
| 18 – 23 | Keep alert for possible increases in the index and for symptoms of heat stress |
| 23 – 28 | Active exercise for unacclimatized persons should be curtailed |
| 28 – 30 | Active exercise for all but the well-acclimated should be curtailed |
| >30 | All training should be stopped |

PET is derived by based on Munich Energy-Balance Model for Individuals (MEMI):

M + W + R + C + E_D_ + E_Re_ + E_Sw_ + S = 0

M: Metabolic rate, W: Physical work output, R: Net radiation of the body, C: Convective heat flow, E_D_: Latent heat flow by diffusion through skin, E_Re_: Latent heat flux due to respiration, E_Sw_: Latent heat flux by evaporation of sweat, S: Storage heat flow

**Table S.4.** Assessment table of PET (Matzarakis et al., 1999)

| **PET (°C)** | **Thermal perception** | **Grade of physiological stress** |
| --- | --- | --- |
| >41 | Very hot | Extreme heat stress |
| 35-41 | Hot | Strong heat stress |
| 29-35 | Warm | Moderate heat stress |
| 23-29 | Slightly warm | Slight heat stress |
| 18-23 | Comfortable | No thermal stress |
| 13-18 | Slightly cool | Slight cold stress |
| 8-13 | Cool | Moderate cold stress |
| 4-8 | Cold | Strong cold stress |
| ≤4 | Very cold | Extreme cold stress |

UTCI is derived by based on UTCI-Fiala model:

M + W + C + K + E + Q + Res ± S = 0

M: Metabolic production, W: Mechanical power, C: Convection, K: Conduction, E: Evaporation, Q: Radiation, Res: Respiration, S: Storage (change in heat content of the body)

**Table S.5.** Assessment table of UTCI (Blazejczyk et al., 2013)

| **UTCI (°C)** | **Stress Category** |
| --- | --- |
| ≥ 46 | Extreme heat stress |
| 38 – 46 | Very strong heat stress |
| 32 – 38 | Strong heat stress |
| 26 – 32 | Moderate heat stress |
| 9 – 26 | No thermal stress |
| 0 – 9 | Slight cold stress |
| -13 – 0 | Moderate cold stress |
| -27 – -13 | Strong cold stress |
| -40 – -27 | Very strong cold stress |
| < -40 | Extreme cold stress |

PT is derived by based on Klima-Michel model:

M - W = (C + R + E_sk_) + (C_res_ + E_res_) + S_sk_ + S_cr_

M: Metabolic heat production, W: Mechanical work, S_sk_: Heat storage in the skin, S_cr_: Heat storage in the core, C: Convective loss of sensible heat, R: Radiative loss of sensible heat, E_sk_: Evaporative loss from the skin, C_res_: Loss of sensible heat by respiration, E_res_: Loss of latent heat by respiration

**Table S.6.** Assessment table of PT (Staiger et al., 2012)

| **PT (°C)** | **Thermal perception** | **Thermo-physiological stress** |
| --- | --- | --- |
| ≥ 38 | Very hot | Extreme heat stress |
| 32 - 38 | Hot | Great heat stress |
| 26 - 32 | Warm | Moderate heat stress |
| 20 - 26 | Slightly warm | Slight heat stress |
| 0 - 20 | Comfortable | Comfort possible |
| -13 - 0 | Slightly cool | Slight cold stress |
| -26 - -13 | Cool | Moderate cold stress |
| -39 - -26 | Cold | Great cold stress |
| < -39 | Very cold | Extreme cold stress |

**Table S.7.** The conditions of sultriness and windchill cases based on Steadman’s tables

| Sultriness case | Windchill case |
| --- | --- |
| 20°C ≤ T_a_ < 23°C and RH > 70% | T_a_ < 29°C and WS > 3 m/s |
| 23°C ≤ T_a_ < 26°C and RH > 60% | 29°C ≤ T_a_ < 33°C and WS > 4 m/s |
| 26°C ≤ T_a_ < 28°C and RH > 50% | 33°C ≤ T_a_ < 34°C and WS > 8 m/s |
| 28°C ≤ T_a_ < 32°C and RH > 40% |  |
| 32°C ≤ T_a_ < 36°C and RH > 30% |  |
| 36°C ≤ T_a_ < 43°C and RH > 25% |  |
| 43°C ≤ T_a_ and RH > 20% |  |


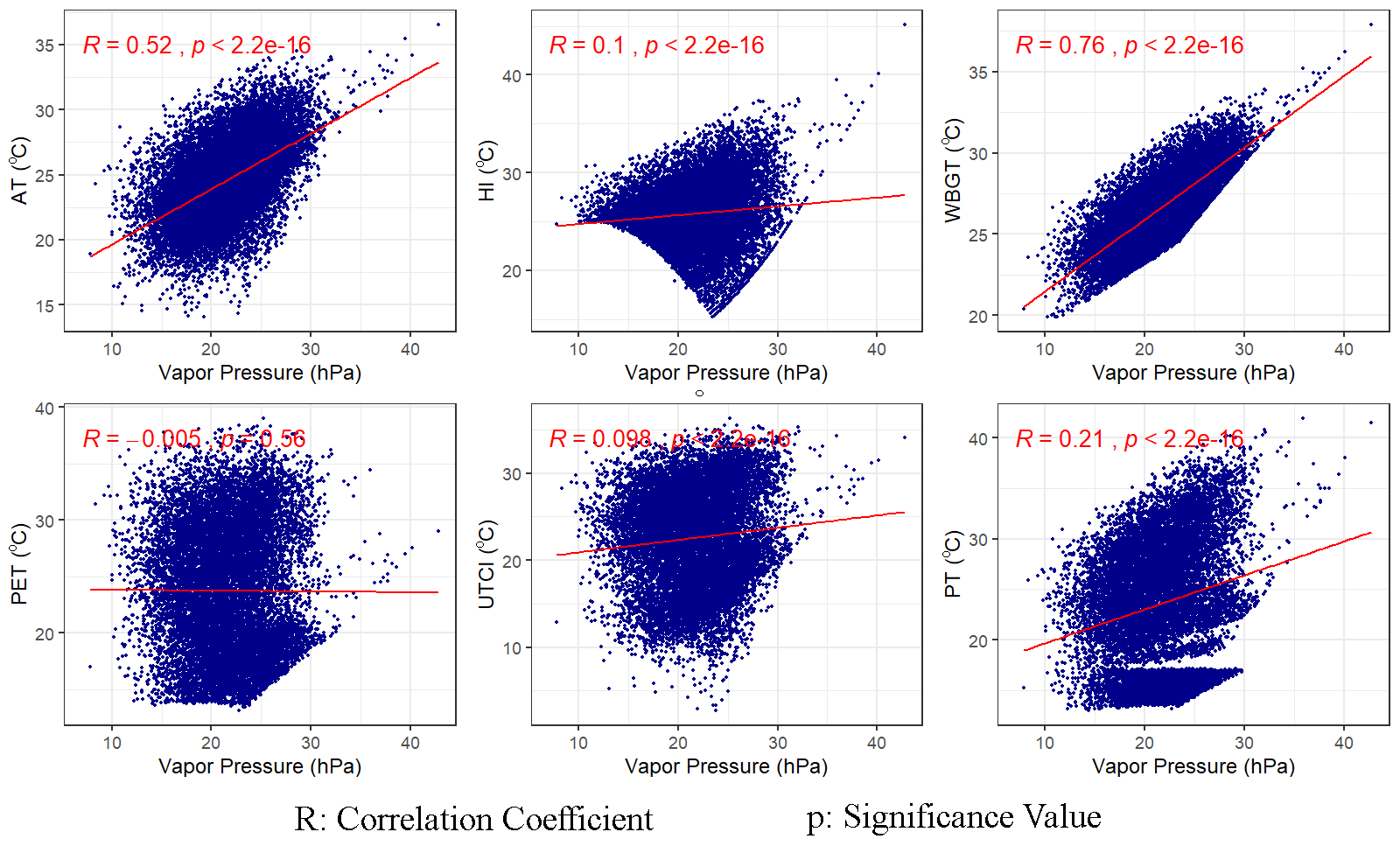


**Figure S.2.** Scatterplot and correlation analysis of vapor pressure and thermal indices

**Results of homogeneity tests for air temperature variable**

Results of Hypothesis Test

--------------------------

Null Hypothesis: Ratio of each pair of variances = 1

Alternative Hypothesis: At least one variance differs

Test Name: Levene's Test for

Homogenity of Variance

Estimated Parameter(s):

Adalar = 6.077783

Arnavutköy = 11.737584

Atatürk Hav. = 11.485790

Beykoz = 13.556342

Beykoz/Ana.Fen. = 9.362033

Büyükçekmece = 18.320192

Çatalca = 10.426970

Çekmeköy = 19.234941

Eyüp = 17.888819

Fatih/Den.Bil. = 14.321280

Fatih/Kumkapı = 8.386869

Florya = 13.802360

Güngören = 12.954484

Kadıköy/Göztepe = 14.007684

Kadıköy/Rıhtım = 12.858479

Kartal = 14.184884

Sab.Gökçen Hav. = 15.218471

Samandıra Hav. = 15.665257

Sancaktepe = 18.209165

Sarıyer = 12.564923

Sarıyer/Kumköy = 14.060686

Silivri = 14.989507

Silivri Fen. = 10.751463

Şile = 14.052138

Şişli = 12.180845

Tuzla = 16.011360

Tuzla/Ayd.Fen. = 8.405260

Tuzla/İTÜ Fen. = 9.009931

Ümraniye = 16.820532

Üsküdar = 12.352540

Data: sicaklik_yaz1

Grouping Variable: Columns

Sample Sizes:

Adalar = 6923

Arnavutköy = 8599

Atatürk Hav. = 11006

Beykoz = 8552

Beykoz/Ana.Fen. = 8272

Büyükçekmece = 10780

Çatalca = 10979

Çekmeköy = 8561

Eyüp = 10442

Fatih/Den.Bil. = 10922

Fatih/Kumkapı = 8782

Florya = 10881

Güngören = 8635

Kadıköy/Göztepe = 7689

Kadıköy/Rıhtım = 10919

Kartal = 10887

Sab.Gökçen Hav. = 10976

Samandıra Hav. = 10594

Sancaktepe = 8157

Sarıyer = 11029

Sarıyer/Kumköy = 11011

Silivri = 8123

Silivri Fen. = 10211

Şile = 10926

Şişli = 8554

Tuzla = 10901

Tuzla/Ayd.Fen. = 10717

Tuzla/İTÜ Fen. = 10485

Ümraniye = 8650

Üsküdar = 8387

Number NA/NaN/Inf's:

Adalar = 4117

Arnavutköy = 2441

Atatürk Hav. = 34

Beykoz = 2488

Beykoz/Ana.Fen. = 2768

Büyükçekmece = 260

Çatalca = 61

Çekmeköy = 2479

Eyüp = 598

Fatih/Den.Bil. = 118

Fatih/Kumkapı = 2258

Florya = 159

Güngören = 2405

Kadıköy/Göztepe = 3351

Kadıköy/Rıhtım = 121

Kartal = 153

Sab.Gökçen Hav. = 64

Samandıra Hav. = 446

Sancaktepe = 2883

Sarıyer = 11

Sarıyer/Kumköy = 29

Silivri = 2917

Silivri Fen. = 829

Şile = 114

Şişli = 2486

Tuzla = 139

Tuzla/Ayd.Fen. = 323

Tuzla/İTÜ Fen. = 555

Ümraniye = 2390

Üsküdar = 2653

Test Statistic: F = 304.1327

Test Statistic Parameters: num df = 29

denom df = 291520

P-value: 0

Results of Hypothesis Test

--------------------------

Null Hypothesis: Ratio of each pair of variances = 1

Alternative Hypothesis: At least one variance differs

Test Name: Bartlett's Test for Homogenity of Variance (With Correction Factor)

Estimated Parameter(s):

Adalar = 6.077783

Arnavutköy = 11.737584

Atatürk Hav. = 11.485790

Beykoz = 13.556342

Beykoz/Ana.Fen. = 9.362033

Büyükçekmece = 18.320192

Çatalca = 10.426970

Çekmeköy = 19.234941

Eyüp = 17.888819

Fatih/Den.Bil. = 14.321280

Fatih/Kumkapı = 8.386869

Florya = 13.802360

Güngören = 12.954484

Kadıköy/Göztepe = 14.007684

Kadıköy/Rıhtım = 12.858479

Kartal = 14.184884

Sab.Gökçen Hav. = 15.218471

Samandıra Hav. = 15.665257

Sancaktepe = 18.209165

Sarıyer = 12.564923

Sarıyer/Kumköy = 14.060686

Silivri = 14.989507

Silivri Fen. = 10.751463

Şile = 14.052138

Şişli = 12.180845

Tuzla = 16.011360

Tuzla/Ayd.Fen. = 8.405260

Tuzla/İTÜ Fen. = 9.009931

Ümraniye = 16.820532

Üsküdar = 12.352540

Data: sicaklik_yaz1

Grouping Variable: Columns

Sample Sizes:

Adalar = 6923

Arnavutköy = 8599

Atatürk Hav. = 11006

Beykoz = 8552

Beykoz/Ana.Fen. = 8272

Büyükçekmece = 10780

Çatalca = 10979

Çekmeköy = 8561

Eyüp = 10442

Fatih/Den.Bil. = 10922

Fatih/Kumkapı = 8782

Florya = 10881

Güngören = 8635

Kadıköy/Göztepe = 7689

Kadıköy/Rıhtım = 10919

Kartal = 10887

Sab.Gökçen Hav. = 10976

Samandıra Hav. = 10594

Sancaktepe = 8157

Sarıyer = 11029

Sarıyer/Kumköy = 11011

Silivri = 8123

Silivri Fen. = 10211

Şile = 10926

Şişli = 8554

Tuzla = 10901

Tuzla/Ayd.Fen. = 10717

Tuzla/İTÜ Fen. = 10485

Ümraniye = 8650

Üsküdar = 8387

Number NA/NaN/Inf's:

Adalar = 4117

Arnavutköy = 2441

Atatürk Hav. = 34

Beykoz = 2488

Beykoz/Ana.Fen. = 2768

Büyükçekmece = 260

Çatalca = 61

Çekmeköy = 2479

Eyüp = 598

Fatih/Den.Bil. = 118

Fatih/Kumkapı = 2258

Florya = 159

Güngören = 2405

Kadıköy/Göztepe = 3351

Kadıköy/Rıhtım = 121

Kartal = 153

Sab.Gökçen Hav. = 64

Samandıra Hav. = 446

Sancaktepe = 2883

Sarıyer = 11

Sarıyer/Kumköy = 29

Silivri = 2917

Silivri Fen. = 829

Şile = 114

Şişli = 2486

Tuzla = 139

Tuzla/Ayd.Fen. = 323

Tuzla/İTÜ Fen. = 555

Ümraniye = 2390

Üsküdar = 2653

Test Statistic: Chisq = 8482.646

Test Statistic Parameter: df = 29

P-value: 0
